# Supplementary figures and images for: A naturally occurring mitochondrial genome variant confers broad protection from infection in Drosophila
Source: PLoS Genet. 2024 Nov 11;20(11):e1011476. doi: 10.1371/journal.pgen.1011476 (PMC11614270; doi:10.1371/journal.pgen.1011476)

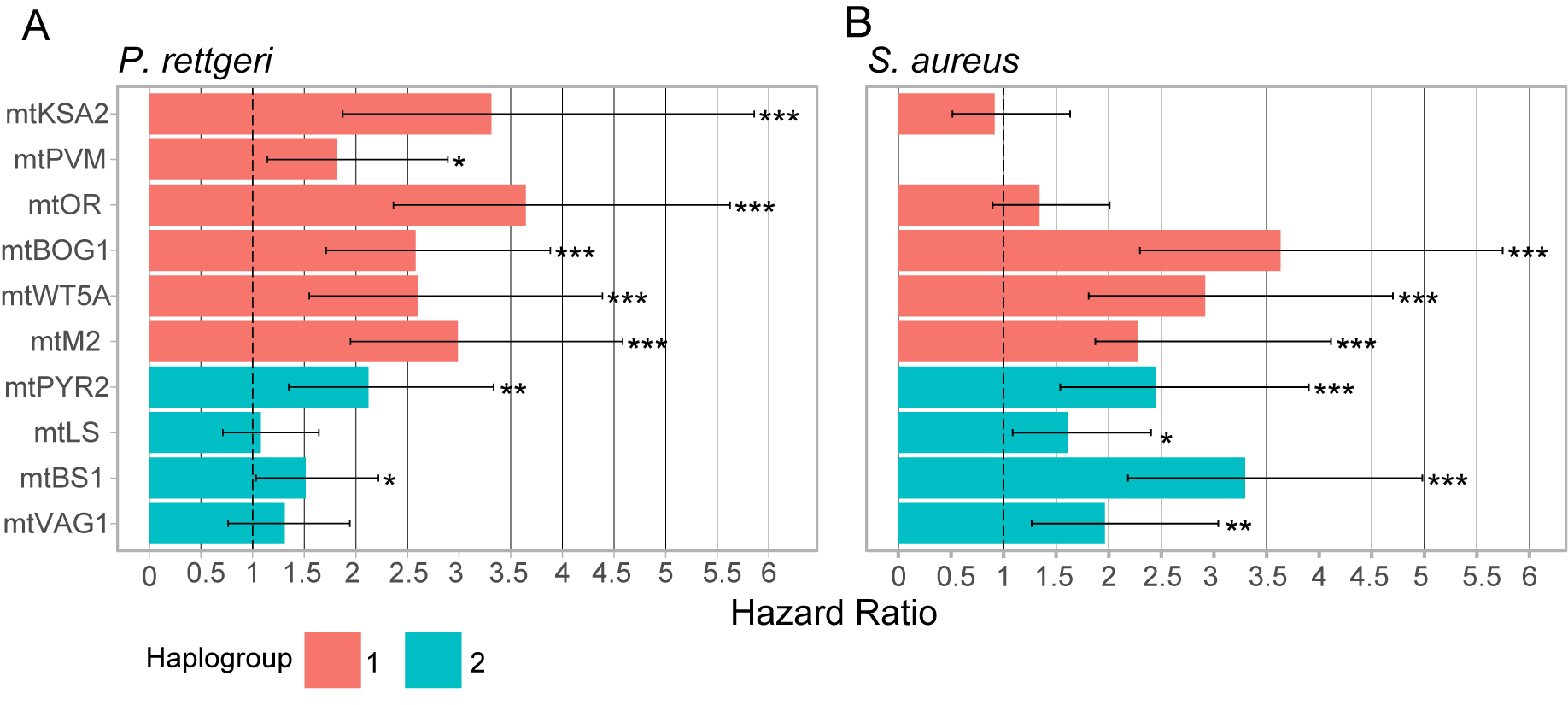

Supplement: S1 Fig — Sex-specific hazard ratios of survivals after (A) P. rettgeri and (B) S. aureus infection. Female to male hazard ratio is calculated based on the results from a Cox-proportional hazard model. Error bars denote 95% confidence intervals. 1 = absence of sexual dimorphism, >1 = greater susceptibility in females. Haplogroups based on [18]. Data was analysed using Log-Rank tests. ns not significant; * p<0.05; ** p<0.01; *** p<0.001. (TIF) [file pgen.1011476.s001.tif]

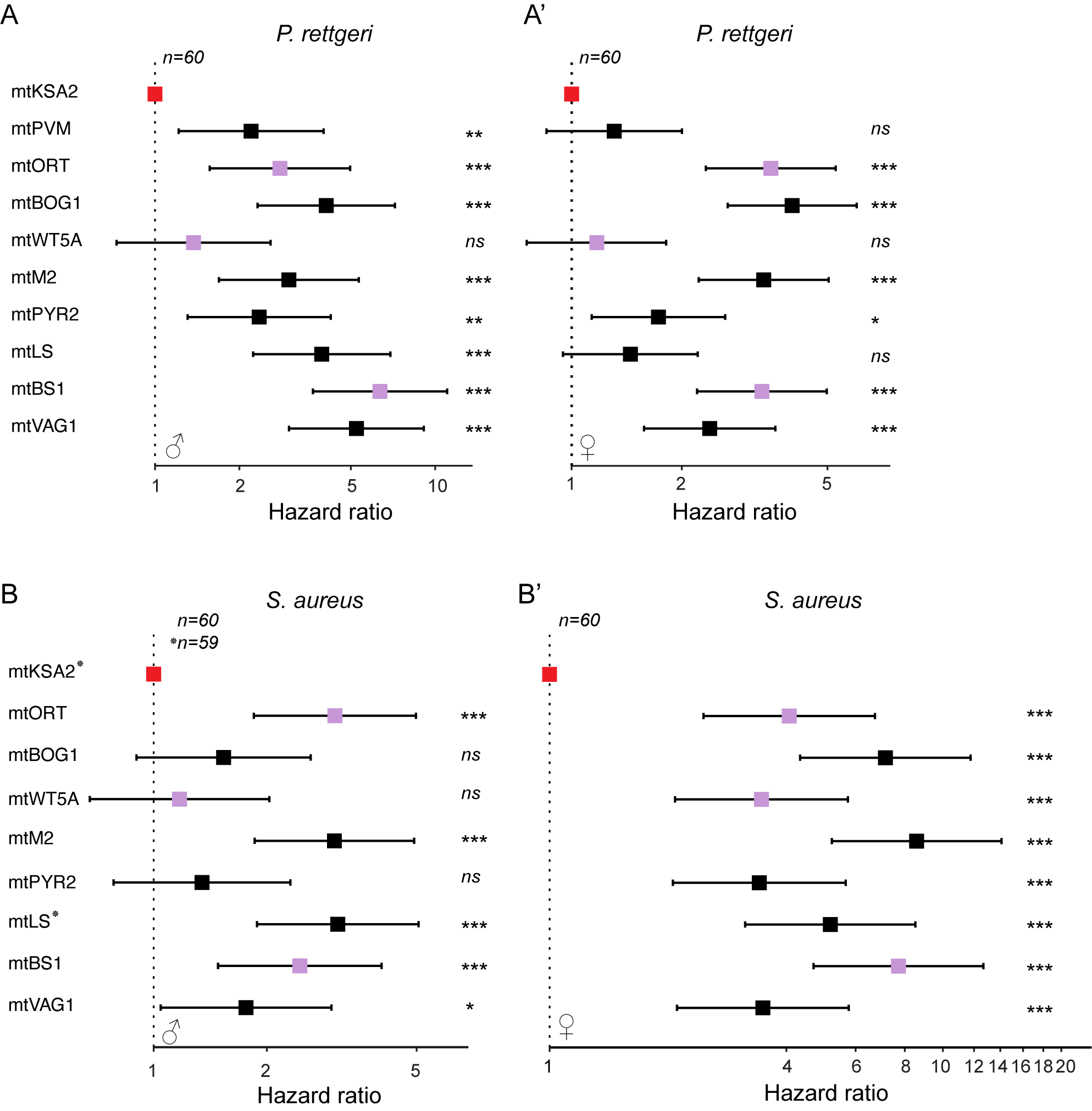

Supplement: S2 Fig — Hazard ratios of survival post (A-A’) P. rettgeri and (B-B’) S. aureus infection with mtKSA2 set as the reference. 1 = absence of mtKSA2 vs. mitotype X variation, >1 = greater susceptibility in mitotype X when compared to mtKSA2. Error bars denote 95% confidence intervals. Data was analysed using Log-Rank tests. ns not significant; * p<0.05; ** p<0.01; *** p<0.001. (TIF) [file pgen.1011476.s002.tif]

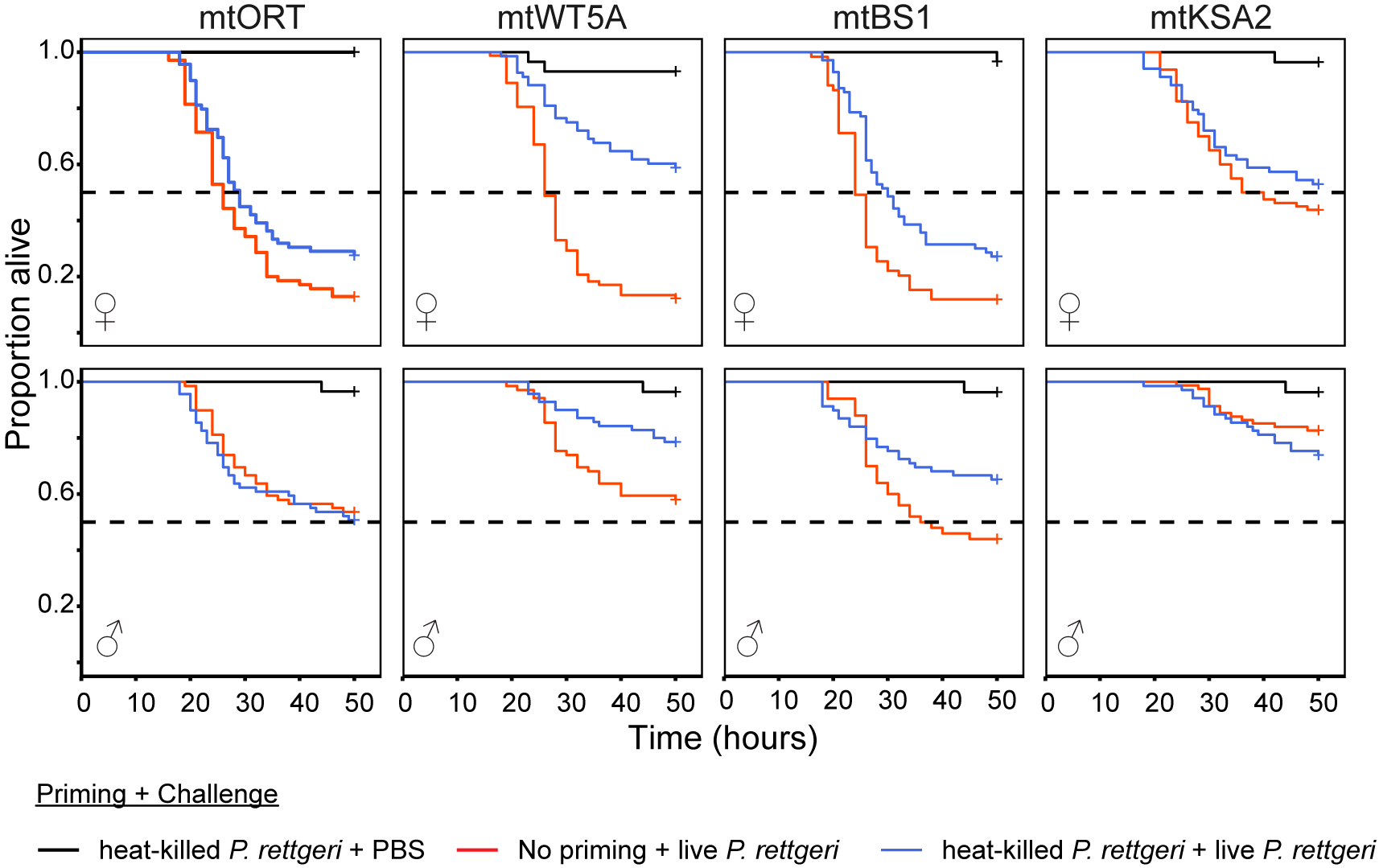

Supplement: S3 Fig — Flies were primed with heat-killed P. rettgeri or left naive and then infected with live P. rettgeri or sham infected with PBS. Survival following priming with the heat-killed pathogen and live challenge differed significantly among mitotypes (Mitotype x Treatment: χ2 = 14.77, p = 0.002) and between males and females (Mitotype x Sex, X2 = 7.553, p = 0.022). Survival data were analysed using Cox Mixed Effects models. (TIF) [file pgen.1011476.s003.tif]

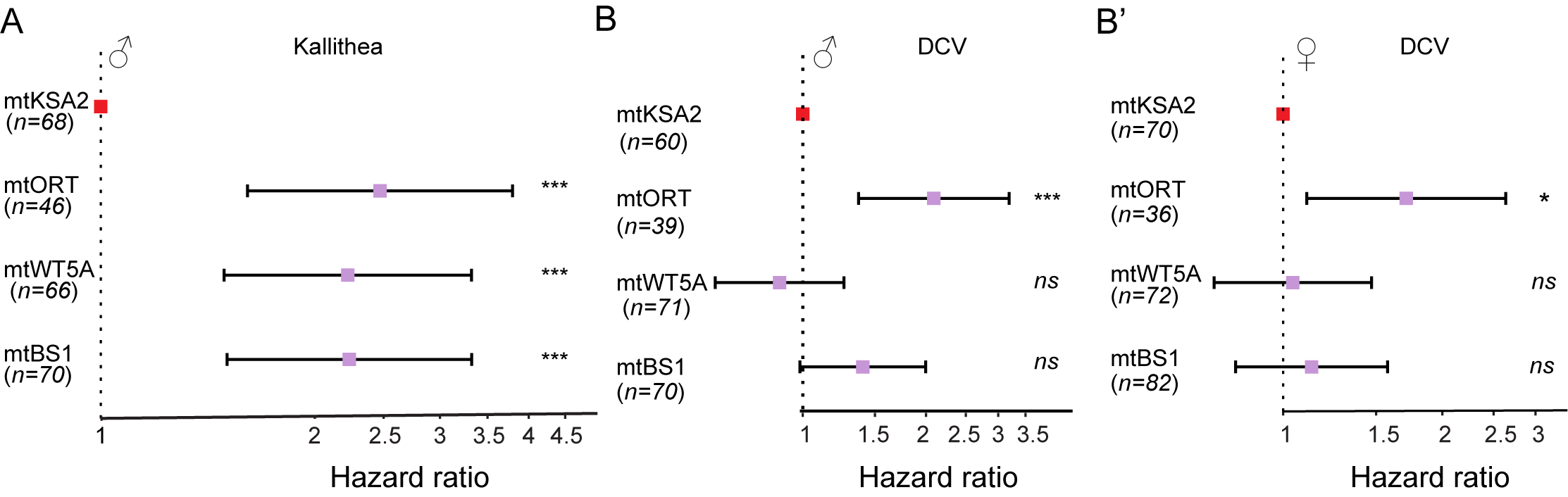

Supplement: S4 Fig — Hazard ratios of survivals after (A) Kallithea and (B-B’) DCV infections with mtKSA2 set as a reference. 1 = absence of mtKSA2 vs. mitotype X variation, >1 = greater susceptibility in mitotype X vs. mtKSA2. Error bars denote 95% confidence intervals. Data was analysed using Log-Rank tests. ns not significant; * p<0.05; ** p<0.01; *** p<0.001. (TIF) [file pgen.1011476.s004.tif]

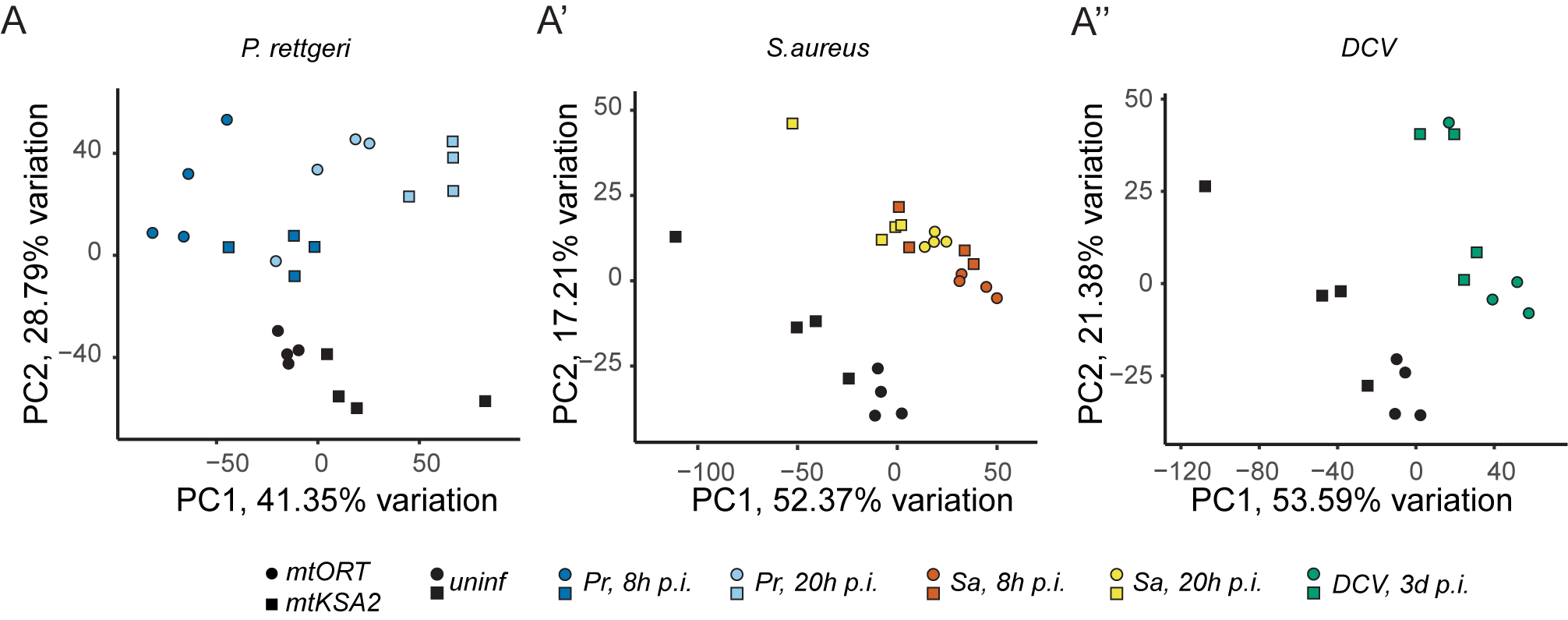

Supplement: S5 Fig — (A-A”) PCA was used to visualize the clustering of the RNA sequencing samples of uninfected and bacterial (A-A’) or viral (A”) infected mtORT and mtKSA2 females. (TIF) [file pgen.1011476.s005.tif]

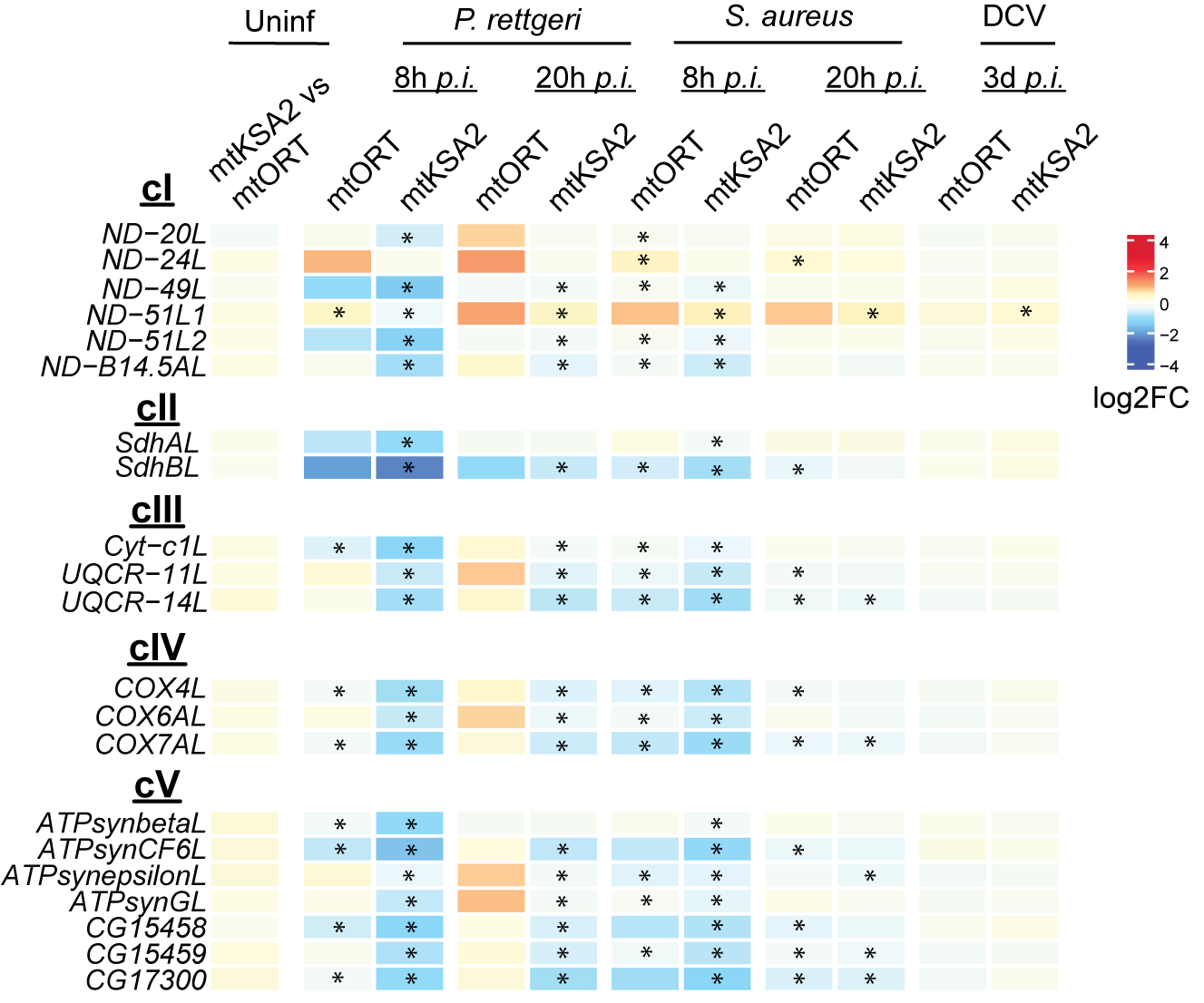

Supplement: S6 Fig — Genes purported to be involved with OXPHOS according to Flybase annotation (http://flybase.org; [50]) were removed from the heatmap in Fig 5 due to their unusual expression patterns. In treatments where other OXPHOS genes were upregulated, the “OXPHOS-like (L)” genes were downregulated and vice versa. (TIF) [file pgen.1011476.s006.tif]

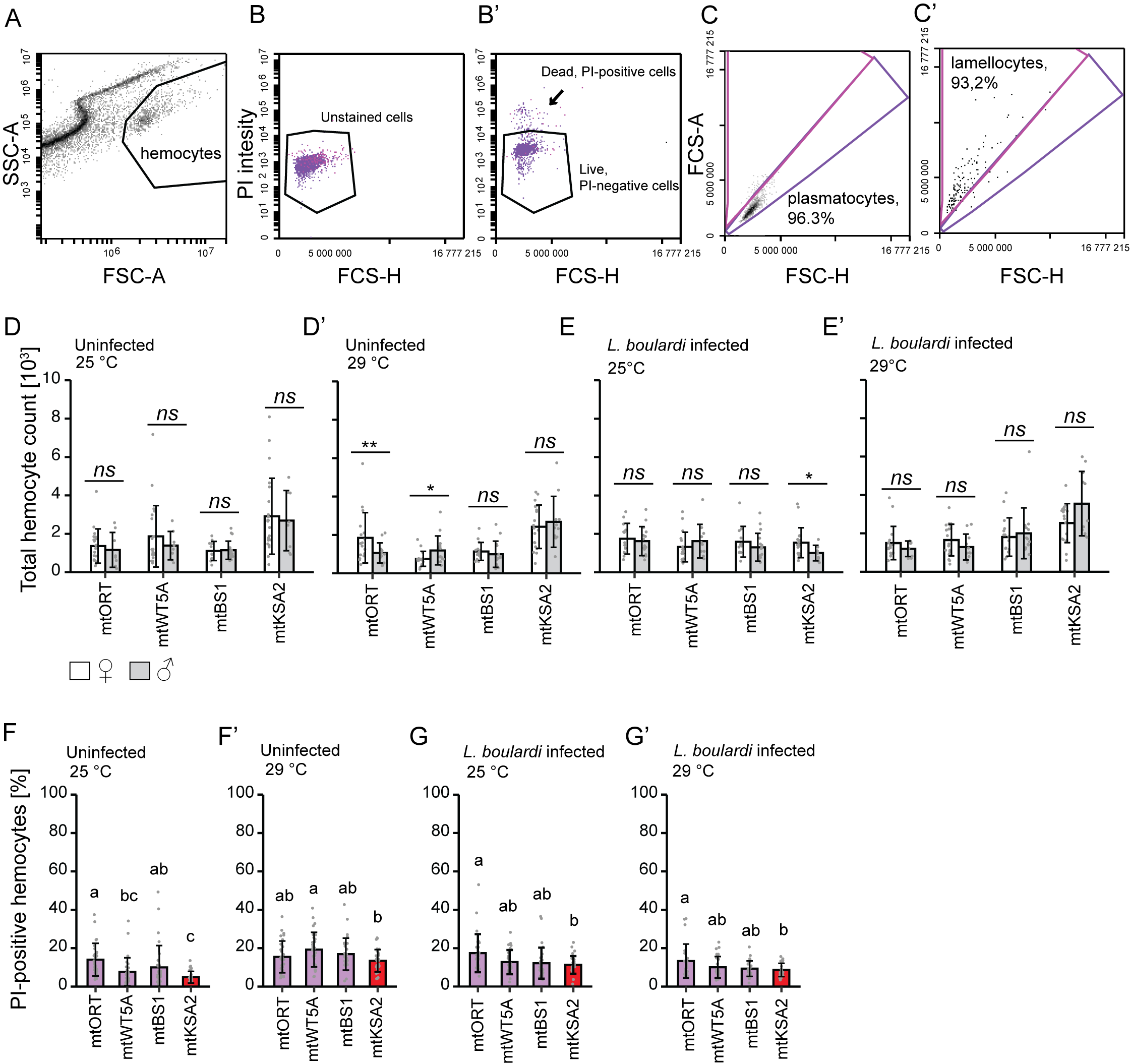

Supplement: S7 Fig — (A) Hemocytes form a population separated from cellular debris based on size and shape in the hemolymph on a forward scatter (FSC-A) vs. side scatter (SSC-A) area plot. Hemocytes in the gated area were used in subsequent analyses. (B-B’) Dead and dying cells become permeable to Propidium iodide (PI) stain, whereas live cells remain unstained. PI was detected using a 488 nm excitation laser and a 610/20 nm emission filter, and the PI signal is shown on the y-axis. The x-axis shows the forward scatter height (FSC-H). (B) Non-stained hemocytes were used to determine where the non-fluorescent cells were located in the plot. An example of the separation of live and dead hemocytes based on the PI-staining is shown in (B’). The live cell gate was used in the subsequent analysis of the total hemocyte counts. (C-C´) Gating strategy to separate steady-state plasmatocytes from activated hemocytes. (C) eaterGFP-positive plasmatocytes from uninfected larvae were gated and plotted in an FSC-A vs. FSC-H plot. The plasmatocytes are found in an approximately 45° angle population due to their round shape. The plasmatocyte gate contains the majority of the eaterGFP -positive hemocytes in the sample. The other area in the plot contains all hemocytes that deviate from plasmatocytes by shape. (C’) msn-mCherry-positive lamellocytes from an infected larva. The majority of lamellocytes fall into the “activated hemocytes” gate. (D-E’) Sex-specific hemocyte counts in (D-D’) uninfected and (E-E’) L. boulardi -infected 3rd instar cybrid larvae, reared at 25°C or 29°C. Female hemocyte counts are shown in white and male hemocyte counts are shown in gray. The data were analysed using a generalized linear model with a negative binomial distribution and temperatures were analysed separately. ns, not significant; *, p < 0.05; ** p< 0.01; ***, p< 0.001. (F-G’) PI-permeable (dead) hemocytes detected in the samples of cybrid larvae. (F-F’) Proportion of dead cells in uninfected cybrid larvae reared at 2 [file pgen.1011476.s007.tif]

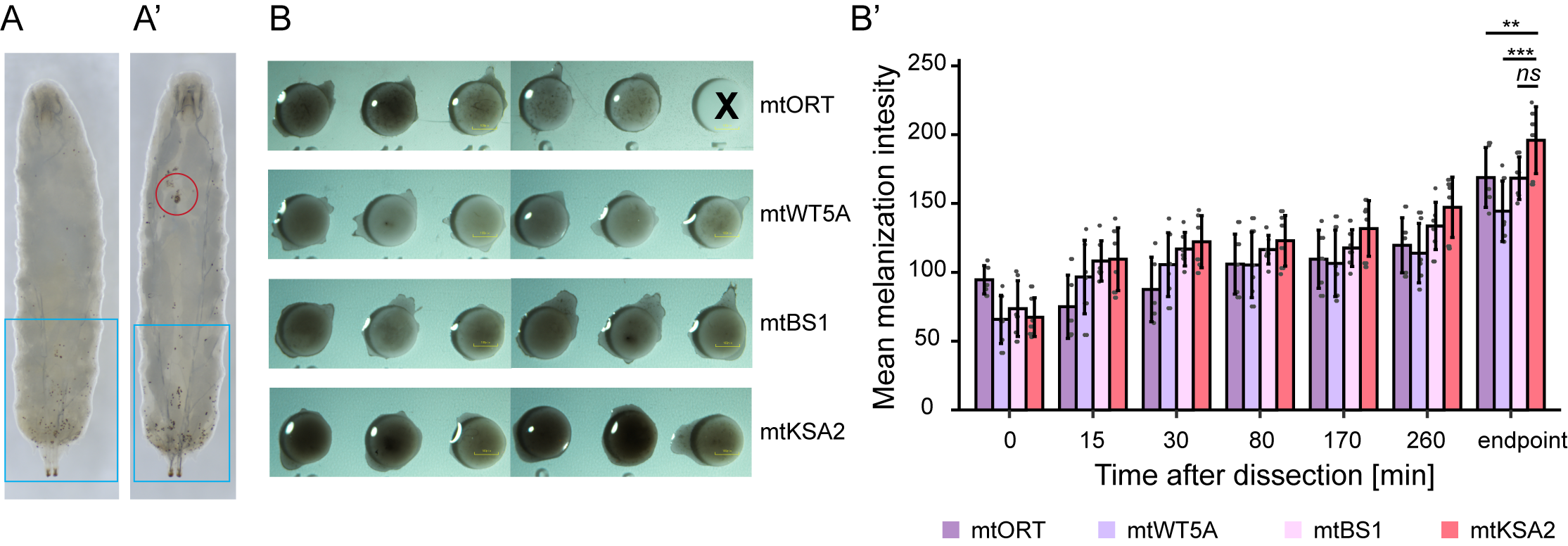

Supplement: S8 Fig — (A) Crystal cells were measured from the three lowest posterior segments from the dorsal site (framed in blue) of the 3rd instar larvae, reared at 29°C. (A’) In some cases, crystal cells were also visible and clustered in the lymph glands (circled in red) of the larvae. (B) Example pictures of hemolymph on glass slides at the endpoint of the experiment, (~ 24 h after dissection). “X” = no sample. (B’) Melanisation intensities measured as mean gray values in each well (excluding the reflection of light seen in some wells). n = 10 for mtORT and n = 12 for the other cybrid lines. Statistical analysis was performed at the endpoint of the experiment using an ANOVA and Tukey’s HSD for pairwise comparisons. Differences of other mitotypes to mtKSA2 marked in the plot. ns not significant; * p < 0.05; ** p< 0.01; *** p< 0.001. (TIF) [file pgen.1011476.s008.tif]

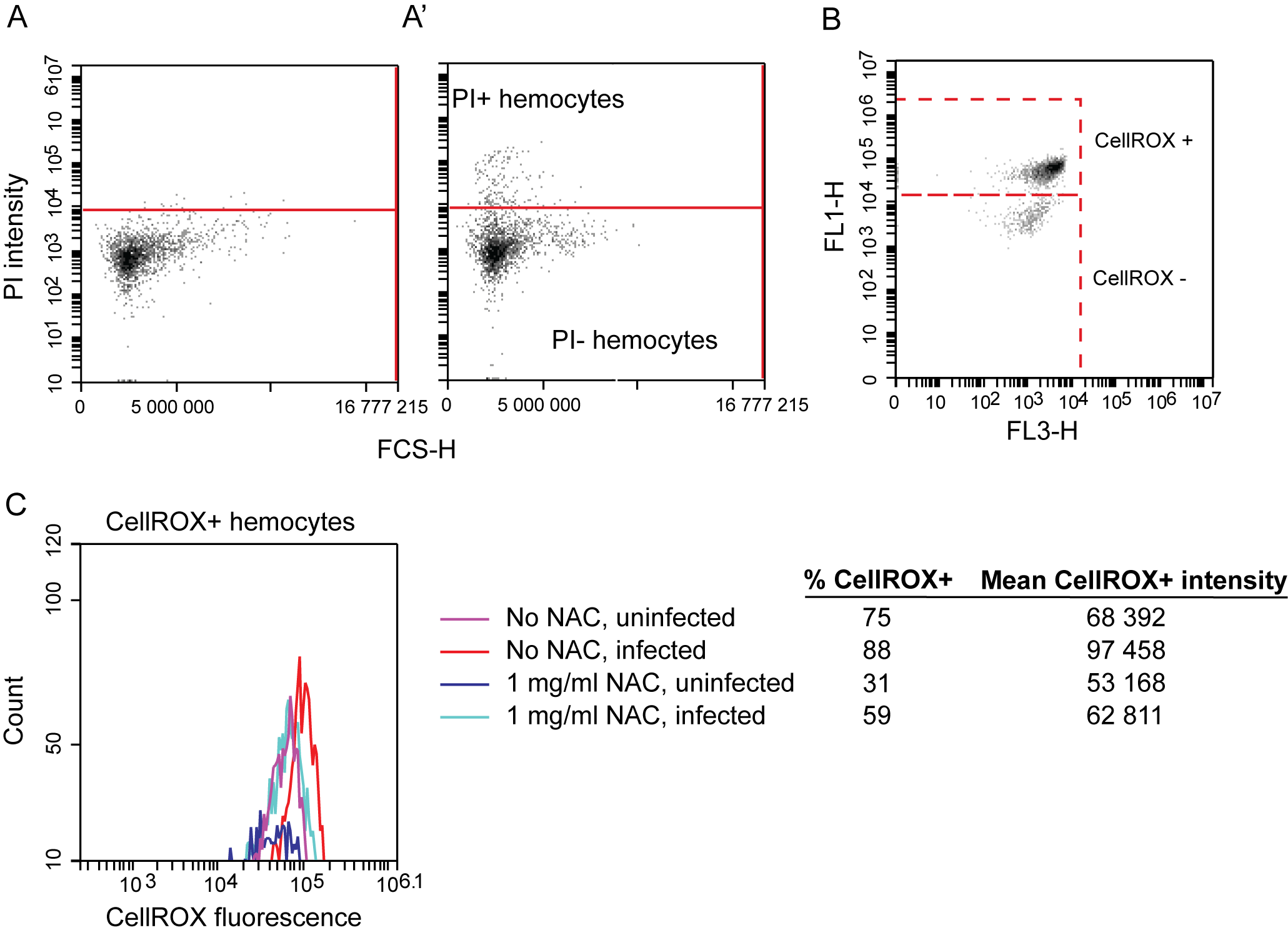

Supplement: S9 Fig — (A) A threshold level was set for the PI intensity based on non-fluorescent hemocytes, and (A’) PI- positive hemocytes were excluded from the analysis. 2000 PI-negative hemocytes were analysed from each sample. (B) CellROX green staining resulted in two populations of hemocytes, a CellROX-positive (CellROX+) and a CellROX negative (CellROX-) population, which could not be separated from the autofluorescence of non-stained hemocytes. (C) Feeding the antioxidant N-acetylcysteine (NAC) to the larvae reduced the proportion of hemocytes falling into the CellROX-positive gate, both in uninfected and in L. boulardi -infected larvae. It also reduced the mean intensity of the CellROX green stain in the CellROX+ gate. (TIF) [file pgen.1011476.s009.tif]
