# Supplementary material for: A naturally occurring mitochondrial genome variant confers broad protection from infection in Drosophila
Source: PLoS Genet. 2024 Nov 11;20(11):e1011476. doi: 10.1371/journal.pgen.1011476 (PMC11614270; doi:10.1371/journal.pgen.1011476)
Supplement: S5 Table — (DOCX) [file pgen.1011476.s014.docx]

**S5 Table.** **mtDNA genome variation among the four cybrid lines based on *D. melanogaster* Reference sequence KT174474.1.** Mitotype specific amino acid replacement variants are found from OXPHOS complexes cIII and cIV. One insertion is found from the *mt:srRNA* and mtDNA length is shown to vary due to the length variation present in the A+T rich non-coding region of the *Drosophila* mitochondrial genome. (Modified from [18]).

| mtDNA | Haplo-group | Origin | Gene Bank no. | *mt:Cyt-b**  (cIII) | *mt:COII**  (cIV) | *mt:COIII**  (cIV) | *mt:srRNA** | mtDNA length  (A+T rich region) |
| --- | --- | --- | --- | --- | --- | --- | --- | --- |
| **mtORT** | I | - | KY559383 |  |  |  | 238insTAA | short |
| mtWT5A | I | USA | KY559387 |  |  |  |  | average |
| mtBS1 | II | Spain | KY559391 |  | G58S |  |  | average |
| **mtKSA2** | I | Zimbabwe | KY559384 | D21N |  | A75T |  | average |

**mt:Cyt-b*= *mitochondrial Cytochrome b*; **mt:COII* = *mitochondrial Cytochrome c oxidase subunit II*; **mt:COII* = *mitochondrial Cytochrome c oxidase subunit III; *mt:srRNA = mitochondrial small ribosomal RNA, 12S rRNA*
