# Supplementary material for: A naturally occurring mitochondrial genome variant confers broad protection from infection in Drosophila
Source: PLoS Genet. 2024 Nov 11;20(11):e1011476. doi: 10.1371/journal.pgen.1011476 (PMC11614270; doi:10.1371/journal.pgen.1011476)
Supplement: S7 Table — (DOCX) [file pgen.1011476.s016.docx]

**S7 Table**. **A list of OXPHOS complex substrates and inhibitors used in respirometry experiment.**

| **Compound** | **Function** | **OXPHOS complex** | **Final concentration** |
| --- | --- | --- | --- |
| Proline | substrate | cI | 5 mM |
| Pyruvate | substrate | cI | 5 mM |
| ADP | substrate/cofactor | cI | 1 mM |
| Rotenone | inhibitor | cI | 600-1500 nM |
| Glycerol-3-phosphate | substrate | cIII | 10 mM |
| Antimycin A | inhibitor | cIII | 60-120 nM |
| Ascorbate | substrate | cIV | 500 µM |
| TMPD* | substrate | cIV | 500 µM |

*Tetramethyl-p-phenylenediamine
