# Supplementary material for: A naturally occurring mitochondrial genome variant confers broad protection from infection in Drosophila
Source: PLoS Genet. 2024 Nov 11;20(11):e1011476. doi: 10.1371/journal.pgen.1011476 (PMC11614270; doi:10.1371/journal.pgen.1011476)
Supplement: S8 Table — (DOCX) [file pgen.1011476.s017.docx]

**S8 Table**. **RT-qPCR primer sequences**

| **Gene*** | **Primer sequence 5’->3’** | **Template** |
| --- | --- | --- |
| *FucTC* | F_ CGCCTGGTTCGTCTCTCATT  R_ GCTGAGTGTTCCGCAGTTTC | Uninfected flies |
| *AANATL3* | F_ GATGAAGTGCGTCCATTCGC  R_ ATAGTGTGTGGTTCTGCGGG | Uninfected flies |
| *Acp1* | F2_ CTGGCCGGACACATCCAGTC  R2_ATACTCCTTACAAGGTTCCGGGTG | Uninfected flies |
| *CG3397* | F_ AGAAGGTGCGTCGTATGGAG  R_ TCGGAGAAGAGCTTGGAGAGA | Uninfected flies |
| *His3.3B* | F_CGCTCAGGATTTCAAGACCG  R_GGATGTCCTTAGGCATGATTG | Control gene. Flies; hemolymph from 3^rd^ instar larvae |
| *16S* | F_TCGTCCAACCATTCATTCCA  R_TGGCCGCAGTATTTTGACTG | mtDNA target gene. mtDNA copy number analysis |
| *RpL32* | F_AGGCCCAAGATCGTGAAGAA  R_TGTGCACCAGGAACTTCTTGAA | nDNA target gene. mtDNA copy number analysis |
| *PPO3* | F_GATGTGGACCGGCCTAACAA  R_CTTAGCGTCATCCAGCACGA | Hemolymph from 3^rd^ instar larvae |

**FucTC* = *alpha1,3-fucosyltransferase C; AANATL3 = Arylalkylamine N-acetyltransferase-like 3; Acp1 = Adult cuticle protein 1; His3.3B = Histone H3.3B; 16S = mitochondrial large ribosomal RNA; RpL32 = Ribosomal protein L32; PPO3 = Prophenoloxidase 3*
