# Supplementary material for: A naturally occurring mitochondrial genome variant confers broad protection from infection in Drosophila
Source: PLoS Genet. 2024 Nov 11;20(11):e1011476. doi: 10.1371/journal.pgen.1011476 (PMC11614270; doi:10.1371/journal.pgen.1011476)
Supplement: Attachment — Submitted filename: Responses to reviewers comments.pdf [file pgen.1011476.s018.pdf]

Dear Editorial Board of PLOS Genetics,

Thank you for your positive handling of our submission and for giving us the opportunity to revise and resubmit our work to PLOS Genetics.

We were very pleased to see that both reviewers – while raising helpful points for improvement – were very positive overall and even broadly complimentary about the motivation, scope, and execution of our work. We thank the two anonymous reviewers for their comments and suggestions for corrections.

Below we offer detailed responses to the reviewers' comments, with the changes marked in purple text in the manuscript ("Salminen et al. manuscript with changes") and indicated with the row line numbers in the response letter.

The main improvements are to the introduction and discussion, which were modified based on the insightful comments by the reviewers. We also took greater care in the description of our statistical analyses and re-analyzed some of the data in line with the reviewer's suggestion to include external sources of variation as random effects in our models. In addition, all the raw data and the R scripts are now available and deposited to Zenodo (https://zenodo.org/records/13378121). This was an unfortunate omission in the initial submission, and possibly the source of many of the questions surrounding the analyses. This should now be clearer to the reviewers.

We hope that you are satisfied with the extent of the revision, which resulted in an improved manuscript.

Sincerely,
Tiina Salminen (on behalf of all co-authors)

Reviewer's Responses to Questions

Comments to the Authors:
Please note here if the review is uploaded as an attachment.

Reviewer #1: This is a really interesting study, comprising a huge amount of work, assaying effects of different naturally occurring mtDNA haplotypes on a range of traits related to the humoral and cell-mediated innate immune response (including but not limited to: survival following bacterial infection, bacterial loads, response to viral infection, RNA sequencing of differential gene expression across all haplotypes and infection statuses, respirometry, ROS production measurement, mtDNA copy number, and an encapsulation response to parasitoid infection).

That's a very impressive amount of work; a credit to the authors that they engaged in such an extensive study. The reporting of results are therefore very detailed, and quite dense (since there is so much to report, and synthesise).

Thank you, we aimed at approaching the study question(s) from multiple different angles.

I think the study and results are very interesting. But I have three major points below that require careful consideration.

1. Conceptual background. I feel the current version of the Introduction is a fairly poorly-developed conceptual synthesis for why mtDNA might be important in immune system regulation. The background provided does not seem to provide a compelling rationale for the study (and given the amount of work the authors have invested, I am sure there is a compelling rationale). The introductory paragraph is framed around the premise that we don't yet understand much of the variation we see in immune system responses, and then pivots immediately into introducing the natural history / genetics of the mitochondrion. This comes across as disjointed – I think it would be better for the authors to spend more time on discussing the factors that might mediate variation in immune responses in invertebrates, and then outline whether some of this variation is genotypic: for example, do we know if immune system variation changes across strains with different nuclear genotype? This would give the rationale for then extending an argument that there might be also good reasons to suspect mtDNA involvement (but you'd need to outline these reasons, and build a rationale). I can think of at least 2 published papers that test for mtDNA-haplotype involvement in immune system function in Drosophila – it would seem important that introducing these form part of your Introduction. An alternative approach would be to build and present evidence for mtDNA involvement in the regulation of innate immune responses (e.g. Fang et al 2016 Protein and Cell, 7:11:16), and then to finish by asking whether variation in mtDNA genotype matters in regulating covariation in immune response function.

We thank the reviewer for pointing this out. Additional reasoning/background for the study is now added to the introduction. Specifically, the role of mitochondria in immune responses is now described on rows 70-72, 75-90, 103-104. We also re-formulated the motivation for the research on rows 145-153 to include previous findings on the mitotype mtKSA2 and to better highlight the reference to Buchanan et al., 2018. We hope these changes help in making the rationale for this study more explicit.

2. Strain creation. The mtDNA strains (cybrid lines) were created by backcrossing (presumably females with the target mtDNAs to males with the isogenic nuclear background – but this is not actually explained) over 10 generations.

Generation of cybrid lines was briefly explained in Materials and Methods section citing the more detailed description of the backcrossing protocol that can be found from Salminen and Vale, 2020 doi: 10.3389/fimmu.2020.00521. More information related to the generation of the cybrid lines was added on rows 722-728. Furthermore, possible drawbacks of the backcrossing method are added to discussion (rows 596-601).

Although this is commonly used, this is not an amazing strategy for partitioning mtDNA from nuclear genetic effects on trait expression (unless various safeguards are built into the design). Ten generations of backcrossing results in replacement of 99.9% of the original nuclear alleles associated with each mtDNA haplotype, placing the target haplotype into a new nuclear background. 0.1% of nuclear genes remain unreplaced; which if looked at the level of unreplaced genes might be 0.1% of 20,000 = around 20 unreplaced nuclear genes. If you look

at the number of unreplaced base pairs, it might be ~ 18000 unreplaced base pairs. The mitochondrial genome only consists of 13 protein coding genes, so there are actually more unreplaced nuclear protein coding genes that will differ across each strain than there are mtDNA genes; and thus it becomes difficult to impossible to conclude whether effects of strains on trait expression are due to the mtDNA effects, or the effects of the unreplaced nuclear genes, or due to other environmental factors that the flies of a particular strain shared (shared environments such as vials etc).

Typically these vial sharing effects / environmental effects are very strong, but the authors don't seem to have accounted for them in their statistical analyses (see point 3). Similarly, uncless backrossing to the isogenic strain continues indefinitely, once the strains are created they will start to accumulate mutations in their nuclear backgrounds over generations, and thus start to diverge from each other in their nuclear genotype across time (creating independent replicates of each strain can help to statistically partition true mtDNA effects from effects of residual nuclear variation across the strains). As a result of the limitation in strain creation, it seems the the results presented here probably provide "some proof of concept" support for the idea that mtDNA haplotype affects immune function via "associations and correlations", but I am not sure the authors can establish causality using this approach; since in theory all the differences across the straits could be due to nuclear genetic variation (unreplaced, or accumulated across the nuclear backgrounds, or even environmental variation across strains).

We agree that the effects originating from the possible residual heterozygosity remaining and/or emerging in the nuclear backgrounds of the cybrid lines cannot be completely ruled out. We added information related to this issue to the discussion as well as previous phenotypic findings related to the effects of mtKSA2 combined with different nuclear backgrounds (rows (rows 574-598). The effects of mtDNA variants are always manifested in cooperation with the present nuclear background. We showed earlier that in some cases the mitovariant mtKSA2 causes similar phenotypic effects on different nuclear backgrounds (Salminen et al. 2017. http://dx.doi.org/10.1016/j.mito.2017.02.001), or drastically different phenotype (synthetic lethality) when compared to other mitovariants on "mitochondrially sick" nuclear background (Salminen et al. 2019 doi: https://doi.org/10.1534/g3.119.400315). Therefore, we are not implying that the mtKSA2 mitovariant would provide immune protection globally (in all nuclear backgrounds), but rather that mtDNA variation leads to variation in infection outcomes and in the case of mtKSA2 in ORT nuclear background the effects are protective against infections.

We have recently linked OXPHOS (dys)function to enhanced immunocompetence of the fly by utilizing RNAi to knock down OXPHOS complex I-V genes in hemocytes (focusing on cIII) (rows 664-672; Vesala et al. 2024 https://doi.org/10.1186/s12915-024-01858-5), providing further evidence for differences in immune traits originating from mitochondria. Studying the effect of natural variation in the mtDNA in an *in vivo* setting is difficult and we acknowledge that further research is required to elucidate the underlying cause in the variation observed in the immune-related traits. But regardless of limitations of the cybrid model, we strongly believe that our work contributes to the understanding of the role of natural mitochondrial variation in immune responses.

3 Statistical methods and inferential pipeline. Simple ANOVAs are used to test effects of

mtDNA haplotype for most traits (and non parametrics alternatives for mtDNA copy number), and mixed models for others (but no details are provided as to the random effects included in these cases). Not enough detail is given regarding the hierarchical structure of the dataset, and therefore it is unclear whether there are random effects that should also be modelled to account for things such as assayed flies sharing the same rearing vials / rearing environments, which if left unaccounted for would result in large levels of pseudoreplication and highly anticonservative results. This sort of structure is typically incorporated via mixed models / multilevel models, which can be conducted in packages like lme4 in R. I think it is really important these things be clarified, and the data and R scripts used made public and referenced in the paper (so readers can find the data and scripts). The authors state the Tukey's tests were calculated, but these are not scaffolded clearly to the descriptions of contrasts between mtDNA haplotypes in the main text of the Results section, or associated figures when the authors are discussing differences in trait values between haplotypes.

We thank the reviewer for pointing out that a more detailed description of the data analyses is in order, and we apologize for not making all of our data and code immediately available - this was an oversight on our part. We have now re-analyzed the data sets when needed (for example, if random effect of vial/replicate was missing) and added more thorough "Statistical analyses" description in the Materials and Methods section (1024-1060) and in the Results section (rows 186-191). We also submitted our code and data to Zenodo (https://zenodo.org/records/13378121). Importantly, re-analysis of the data did not change conclusions made based on the data nor did it change the significance levels reported in the Figures.

While going through the statistics, we decided on making a couple of other changes as well:

1. The data on ROS (Figure 6B-B') was re-analyzed using linear models and corrected for multiple testing using a Holm correction, instead of multiple K-W tests. This did not change the interpretation, nor the significance levels reported.

2. Regarding the sex-differences in hemocyte counts (S7D-E' Fig.) we opted for following the GLM with negative binomial distribution (hemocyte data tend to be over dispersed) with the "pairs" command in the Emmeans package for pairwise comparison of sexes within a mitotype. By doing this, we now obtained previously undetected significant differences in hemocyte counts between the sexes in uninfected mtORT and mtWT5A at 29 °C (S7D' Fig.) and in mtKSA2 after *L. boulardi* infection at 25 °C (S7E Fig.), while the previously significant difference in mtKSA2 after *L. boulardi* infection at 29°C (S7E' Fig.) was no longer statistically significant. Because these few sex-specific differences were not consistent and they were quite small, and because sex-differences were not our focus here, the analysis of the main hemocyte data (Fig. 8B-C') was left as it was (analyzed from pooled male & female samples). Text was modified accordingly on lines 477-478.

3. To analyze the proportion of PI-positive (dead) hemocytes (S7F-G' Fig.) between the mitotypes we used pairwise contrasts using the Emmeans package. This did not have a major effect on the results. However, in S7F Fig., we uncovered a significant difference between mtORT and mtWT5A, in addition to the differences found previously between mtORT vs mtKSA2 and mtBS1 vs mtKSA2. In S7G Fig., only difference between mtORT and mtKSA2 remained

significant. We also edited the plots to resemble their counterparts in Fig. 8B-C' and did small modifications to the text on lines 485-486.

4. There was a mistake in S8B' Fig.: the plot indicated that the melanisation response of mtKSA2 was significantly different when compared to any other mitotype. Although there is more melanisation in mtKSA2 than in other mitotypes, the difference to mtBS1 is not statistically significant. The text was modified on lines 505-506 and 707-708.

Transcriptomic Data: I am not an expert on how these are best analysed and presented; but it appears the authors have generated lists of differentially expressed genes, with FDR-correction, and then perhaps made a lot of contrasts between these lists for different haplotypes in different states of bacterial / virus infection. Are these contrasts made using a statistical approach (akin to Tukey's contrasts), or just by comparing values of expression for genes across different haplotypes / states. Given the very high number of contrasts / comparisons made here, there is a very high chance of Type 1 errors of inference, an thus it seems important that there be some means of correcting for the number of tests / comparisons being made from the RNA sequencing results. These datasets are often analysed using general linear mixed models I believe, rather than the approach that seems to be used here. Also, why was a FDR of $p < 0.15$ used here. Is there are precedence for this – isn't a FDR p value expected to be much lower than 0.05?

The statistical analyses of RNA sequencing data were performed at Edinburgh Genomics, the Genomics and Bioinformatics core facility at the University of Edinburgh and followed their standard pipeline. Additional description of the analyses was added to the Materials and methods section (rows 842-845) based on the report we received from Edinburgh Genomics.

The selection of the FDR p-value depends largely on what one wants to get out of the data. If false positives are absolutely to be avoided, one can select a strict p-value cut off at 0.05 or even 0.01. Because our aim in the current manuscript was to obtain a more general view on which processes, pathways and gene groups might be affected by mitotype we opted for more relaxed FDR of $p<0.15$.

Reviewer #2: In this manuscript, the authors study how mtDNA-nuclear DNA interactions affect immune responses. The authors exposed several isogenic fly lines with identical nuclear genetic background (Oregon RT) but different mitochondrial genomes to various infections. The authors identified the mtKSA2 mtDNA haplotype as particularly resistant to infections, showing an improved survival response to bacterial and viral infections. The authors also demonstrate that the mtKSA2 fly line presents with an increased hemocyte count and an enhanced cell-mediated innate immune response at the larval stage that potentially aids in killing parasites.

This is an ambitious manuscript with clear observations regarding the immune phenotypes. However, the manuscript does not provide a clear mechanism for how the different mtDNA haplotypes affect the immune response. Nevertheless, the manuscript describes an interesting and important observation.

Thank you for finding our work ambitious, and that we describe an important phenomenon that we believe will receive interest in the fields of mitochondrial genetics, immunology and infection biology.

Besides the immunophenotyping the manuscript mainly describes data from various RNA sequencing analysis. The authors should be aware that albeit RNAseq is an important tool to understand differences in cellular responses, especially in mitochondrial biology, the proteome, and even post-translational modifications, are not always reflected in RNAseq data. This might be one of the reasons the authors do not reproduce the observed changes for mitochondrial activity or TMRM levels. Thus, despite the initial observation a mechanism connecting mtDNA haplotype, mitochondrial function, and immune response, is missing.

We acknowledge that the gene expression patterns do not always reflect the proteome level changes and we had briefly mentioned this in the manuscript, now transferred to lines 612-616.

The authors propose that a variant in mt:cytb is responsible for the improved immune response, due to a mild CIII defect increasing hemocyte count. Ideally, mtDNA editing in mt:cytb could be applied, to isolate the individual mtDNA variant, but I accept that this is beyond the scope of this manuscript. However, the authors might want to consider measuring CIII activity in isolated hemocytes to strengthen their hypothesis.

We recently showed (Vesala et al. 2024 https://doi.org/10.1186/s12915-024-01858-5) that knocking down nuclear encoded subunits of OXPHOS complexes hemocyte specifically causes immune activation of hemocytes and enhances the cell-mediated innate immune response against parasitoid wasps. In Vesala et al. we focused on complex III gene *UQCR-C1* as we had earlier noticed (Salminen et al. 2019 doi: https://doi.org/10.1534/g3.119.400315) that mtKSA2 mitotype (with the cIII mt:cytb variant) as well as complex III inhibitor Antimycin A led to the formation of melanotic nodules (melanized hemocyte aggregates that are an indicator of aberrantly activated cell-mediated innate immune response) in larvae and adults. However, when the same OXPHOS gene knockdowns were performed fat body specifically, the immune responses of the hosts were weaker, as the treatment was too drastic for the metabolism of the fly. We hypothesize that mild enough alterations of OXPHOS (not just cIII, but other OXPHOS complexes as well) at the whole fly level (such as specific inherited mtDNA mutations) might prove to be beneficial upon certain situations, such as when exposed to pathogens, and that these responses most likely are tissue specific.

Comments:

1. The authors routinely refer to mtDNA affecting a certain response (e.g. page 6, first sentence) “Furthermore, mtDNA drastically affected the survival response of the hosts after P. rettgeri infection ..."), presumably refering to the specific mtDNA haplotype or mitotype. I suggest rephrasing this so that it is not confused with mtDNA copy number.

We rephrased the sentences where we refer to effects of different mitotypes or mitovariants (instead of using the term mtDNA).

2. Could the authors please explain in the text how they calculate the survival response. Is it based on the assumption that 100% of the uninfected lines will survive?

Survival was measured as flies alive at the time at check points (i.e., time to event, Figure 1A-B'; Figure 2) throughout the course of an experiment (until 60 h post *P. rettgeri* infection, 120 h post *S. aureus*, 14 days post DCV and 24 days post Kallithea infection). The data on survived/dead files at each checkpoint is the basis for survival curve analyses using the Mantel-Cox (Logrank) test which was applied to compare the survival curves of the mitotypes to check if there are any (overall) differences in the survival from infection among the mitotypes. In bacterial infection experiments, the flies are quite young (around 4.5-9 days) at the end of the experiment, so the survival of uninfected flies is assumed to be close to 100%. Kallithea and DCV infections take longer to cause mortality, and in these cases, we included treatments where we injected (control for Kallithea) or pricked (control for DCV) flies with the buffer used in the viral titrations as a sham/ wounding control (Figure 2 A-B', dashed lines).

3. To this reviewer, the mtORT line seems the most suitable control line for comparisons, as this line has undergone expensive natural selection, and other mtDNA haplotypes being tested against this background. Could the authors explain in the text more clearly why they chose a different approach for comparisons?

After finding that there is a statistically significant overall difference in the survival rates among the mitotypes and that mtKSA2 seemed to survive better than any other mitotype (Fig. 1 A-B'), our interest was directed to finding out if mtKSA2 survival was indeed the best. Therefore, we performed the hazard ratios using the mtKSA2 as a baseline and indeed nearly all other mitotypes performed worse in comparison to mtKSA2 (SFig 2). In the experiments where we used the four mitotypes (mtORT, mtWT5A, mtBS1 and mtKSA2) we did not specifically treat any of them as a "control".

4. It is not clear in Figures 1 A, A', B and B' what the statistical significance refer to. What comparisons are performed and what do the significance stars refer to? Are all compared to mtKSA2? Please specify in the manuscript.

Overall differences in the survival among the mitotypes were analysed using the Mantel-Cox log rank test, showing that mitotype cause variation in the survival response. The null hypothesis for the test is that there is no difference in the survival rates between the strains being compared. We further tested the survival rate of mtKSA2 in comparison to the other mitotypes, shown in Supplementary Figure 2. We modified the caption in Figure 1A to clarify the matter.

5. I recommend presenting the differences between groups in Figures 3A´-A´´ more clearly. The colours are very similar and the individual symbols too small to distinguish.

We have now selected new colors with clearer contrast to each other and enlarged & outlined the symbols in 3A’-A’’’ (just two symbols have been used, circles and squares) to aid distinguishing them from each other.

6. In Figure 3D (qRT PCR results) the spread for some samples is very large with a small sample number. I recommend increasing the sample size to improve the data, as no conclusion can currently be made from the data. (e.g. lines mtKSA2 mtWT5A).

qRT PCR data is not as accurate as the one obtained with the RNA sequencing. However, the expression patterns of the few selected genes in Figure 3D have similar expression patterns between mtKSA2 and mtORT in the qRT PCR data as in the RNAseq. For the qRT PCR we collected new set of samples and they showed more variation than the ones included in RNAseq. As explained below (point 8), mtWT5A and mtBS1 were added to the qRT PCR analysis to give more indication of the general expression patterns of the selected genes and whether certain expression patterns are specific for mtORT (susceptible) or mtKSA2 (enhanced immunocompetence), or for the more resistant/tolerant strains (mtKSA2 and mtWT5A) when compared the more susceptible strains (mtORT and mtBS1), or more random expression patterns among the four mitovariants.

7. A Western blot of your transcript candidates would also be helpful.

As we did not find anything especially interesting related to the expression patterns of the selected genes (in Figure 3D), we decided not to study the role of these genes further, e.g. by performing a western blot analysis.

8. Please explain in the manuscript why mtWT5A was included in Figure 3D.

mtWT5A was included in the Figure 3D for the same reason as mtBS1. The reasoning was mentioned in the text (rows 288-292): “When comparing the transcriptomes of just two mitotypes, we could not conclude if the genes of interest are specifically up- or downregulated in the mitotype of interest. Hence, we measured the expression patterns of the representative genes with RT-qPCR from uninfected mtORT and mtKSA2, and additionally from mtBS1 and mtWT5A, with a new set of male samples.”

9. A second experiment supporting the increased ROS production in mtKSA2 lines, such as aconitase activities, would be suitable.

It is certainly true that there are many different approaches for measuring the levels of ROS, aconitase activity assay being one of these. In the current manuscript we studied the role of ROS using three approaches: **1)** the expression patterns of genes functioning in ROS production and scavenging based on the RNA sequencing data of adult flies (Fig. 6A), **2)** hydrogen peroxide content of the whole fly extracts measured fluorometrically with the Amplex Red Hydrogen peroxide/ Peroxidase assay kit (Fig. 6B-B’), and **3)** hemocyte-specific ROS in larval hemocytes, by utilizing the widely used fluorogenic probe CellROX for measuring

oxidative stress of living cells with flow cytometry (Fig. 8G). By using these methods, the tissue specific approach (Fig. 8G) showed that mtKSA2 hemocytes contain more ROS, whereas the ROS measurements that based on the whole fly samples (Fig. 6) did not reveal major differences between mtKSA2 and the other mitovariants.

10. A more sensitive way to analyse OXPHOS function would be to measure isolated OXPHOS activities spectrophotometrically. In this way, even a mild CIII defect could be identified.

This would be a good alternative for respiration-based measurements. Unfortunately, we don't have this method up and running and therefore including those experiments is out of the scope of the current manuscript. In addition, a possible mild vs. more severe cIII defect could be present only in specific tissues of the fly, and in whole animal samples it could be masked by the presence of other tissues with a different response to the mutation of interest. As the strongest immune phenotype was found in the hemocytes, that tissue is most likely where the cIII defect would play a role in the immune response variation we observe. Unfortunately, we do not currently have the means of collecting adequate amount of hemocytes for respiration or spectrophotometry-based protocols.

11. The authors use both stars (*) and lettering (a,b etc) to indicate statistical significance. I understand there is thought put behind this, but it becomes a bit difficult to understand the different approaches. Could the authors just use one approach throughout the manuscript?

In the occasions where the statistical significance has been marked with letters (Figure 8), pair-wise comparisons were made between all the four strains presented (as none of them is a control strain in a sense). We'd prefer to leave them as they are, since marking all pair-wise comparisons with stars would make the plots look quite crowded.

Have all data underlying the figures and results presented in the manuscript been provided?
Large-scale datasets should be made available via a public repository as described in the *PLOS Genetics* data availability policy, and numerical data that underlies graphs or summary statistics should be provided in spreadsheet form as supporting information.

Reviewer #1: Yes

Reviewer #2: Yes

PLOS authors have the option to publish the peer review history of their article (what does this mean?). If published, this will include your full peer review and any attached files.

If you choose "no", your identity will remain anonymous but your review may still be made

public.

Do you want your identity to be public for this peer review? For information about this choice, including consent withdrawal, please see our Privacy Policy.

Reviewer #1: No

Reviewer #2: No
